# Allelic Origin of Protease-Sensitive and Protease-Resistant Prion Protein Isoforms in Gerstmann-Sträussler-Scheinker Disease with the P102L Mutation

**DOI:** 10.1371/journal.pone.0032382

**Published:** 2012-02-23

**Authors:** Salvatore Monaco, Michele Fiorini, Alessia Farinazzo, Sergio Ferrari, Matteo Gelati, Pedro Piccardo, Gianluigi Zanusso, Bernardino Ghetti

**Affiliations:** 1 Department of Neurological, Neuropsychological, Morphological and Motor Sciences, University of Verona, Verona, Italy; 2 Center for Biologics Evaluation and Research, Food and Drug Administration, Rockville, Maryland, United States of America; 3 Department of Pathology and Laboratory Medicine, Indiana University School of Medicine, Indianapolis, Indiana, United States of America; Nagasaki University Graduate School of Biomedical Sciences, Japan

## Abstract

Gerstmann-Sträussler-Scheinker (GSS) disease is a dominantly inherited prion disease associated with point mutations in the *Prion Protein* gene. The most frequent mutation associated with GSS involves a proline-to-leucine substitution at residue 102 of the prion protein, and is characterized by marked variability at clinical, pathological and molecular levels. Previous investigations of GSS P102L have shown that disease-associated pathological prion protein, or PrP^Sc^, consists of two main conformers, which under exogenous proteolysis generates a core fragment of 21 kDa and an internal fragment of 8 kDa. Both conformers are detected in subjects with spongiform degeneration, whereas only the 8 kDa fragment is recovered in cases lacking spongiosis. Several studies have reported an exclusive derivation of protease-resistant PrP^Sc^ isoforms from the mutated allele; however, more recently, the propagation of protease-resistant wild-type PrP^Sc^ has been described. Here we analyze the molecular and pathological phenotype of six GSS P102L cases characterized by the presence of 21 and 8 kDa PrP fragments and two subjects with only the 8 kDa PrP fragment. Using sensitive protein separation techniques and Western blots with antibodies differentially recognizing wild-type and mutant PrP we observed a range of PrP^Sc^ allelic conformers, either resistant or sensitive to protease treatment in all investigated subjects. Additionally, tissue deposition of protease-sensitive wild-type PrP^Sc^ molecules was seen by conventional PrP immunohistochemistry and paraffin-embedded tissue blot. Our findings enlarge the spectrum of conformational allelic PrP^Sc^ quasispecies propagating in GSS P102L thus providing a molecular support to the spectrum of disease phenotypes, and, in addition, impact the diagnostic role of PrP immunohistochemistry in prion diseases.

## Introduction

Prion diseases are fatal neurodegenerative disorders of humans and animals, occurring as idiopathic, genetic, and iatrogenic conditions [1]. Human prion disorders include Creutzfeldt-Jakob disease (CJD), variant Creutzfeldt-Jakob disease, kuru, fatal familial insomnia, sporadic fatal insomnia, and Gerstmann-Sträussler-Scheinker (GSS) disease [2]. In these disorders, the cellular prion protein, or PrP^C^, is converted to a protease-resistant conformer with abnormal sedimentation properties, or PrP^Sc^ [3]. In some prion diseases variant PrP^Sc^ discloses distinct proteolytic sites generating a core fragment named PrP27-30 [4], reflecting the tertiary structure of PrP^Sc^, in addition to variable C-terminal truncated fragments (CTFs) and internal fragments [5], [6], [7], [8], [9]. PrP^Sc^ conformers are classified on the basis of the glycosylation status and the molecular mass of their protease-resistant unglycosylated backbone, or PrP27-30 core fragment, migrating at 21 kDa in type 1 PrP^Sc^ and at 19 kDa in type 2 PrP^Sc^ [10], although controversial results have been obtained by different authors who have proposed the classification of PrP27–30 into four types [11]. However, several prion strains, either in naturally occurring disorders or in experimentally-induced conditions, harbour protease-sensitive PrP^Sc^ (sPrP^Sc^) quasispecies, in association or not with protease-resistant PrP^Sc^ (rPrP^Sc^) [12], [13], [14]; importantly, sPrP^Sc^ molecules cause neurological dysfunction [15], [16]. The neuropathological hallmarks of prion diseases are prion protein (PrP) deposition, spongiosis, astrogliosis, and neuronal death [17]; however, it is still unknown which molecular forms of PrP^Sc^ are detected by immunohistochemistry (IHC).

GSS is an autosomal dominant prion disease associated with point mutations in the *Prion Protein* gene (*PRNP*). The most frequent mutation associated with GSS involves a proline-to-leucine substitution within the unstructured N-terminal region of the PrP at codon 102 (P102L), segregating with methionine at codon 129 on the mutant allele [18]. Other mutations have been described either at the N-terminal or at the C-terminal PrP region. The genetic heterogeneity of GSS partly explains the variability in age of onset, disease duration, and the ample number of clinical variants. However, phenotypic heterogeneity is also observed both within and between pedigrees with the same mutation, especially in P102L GSS cases [19], [20], [21]. Accordingly, while in some P102L patients progressive gait disturbances, truncal ataxia, lower limb hyporeflexia and dysarthria are the presenting clinical features, followed over years by dementia and pyramidal signs, in other cases a rapid evolving dementia, indistinguishable from sporadic CJD (sCJD), is observed. As yet, the molecular basis for such clinical variability remains to be fully explained. Neuropathological examination in GSS P102L discloses marked variation in the degree and brain regional distribution of spongiform changes, which largely correlates with the biochemical detection of proteinase K (PK)-resistant PrP^Sc^ with a core fragment of 21 kDa [8], [9], [22].

As opposed to the variable topographical distribution of spongiform degeneration, typical multi-centric plaques are widely distributed throughout cerebral and cerebellar cortical and subcortical regions, and appear to be constituted by an abnormal PrP isoform that upon PK-digestion generates an internal 8 kDa fragment (PrP8), overall spanning from residues 74–90 to 146–153; this fragment is natively present in brain extract of GSS P102L, although is enriched after PK treatment. Experimental transmission of GSS P102L brain tissues with only PrP8 induces PrP-amyloid deposition, but not spongiform encephalopathy [23]. Earlier studies have shown that abnormal PrP isoforms detected in P102L mutation derive from mutated PrP (PrP^M^) [8]. Recently, this view has been challenged and proof has been obtained of wild-type PrP (PrP^Wt^) involvement in three GSS P102L cases with the 21 kDa fragment [24]. Additionally, evidence has been provided that in transgenic (Tg) mice overexpressing the P101L mutation [Tg,(*prnp*,P101L)], corresponding to GSS P102L mutation, spontaneous formation of mutant sPrP^Sc^ molecules occurs in brain tissues [15], [16]. Moreover, in Tg,(PrP,P101L) expressing high levels of mutant PrP, spontaneous prion disease is accompanied by generation of sPrP^Sc^ [14]. Endogenous generation of sPrP^Sc^ was also observed in Tg196 mice expressing lower levels of P101L mutated protein (about 2-fold higher than wild type), following inoculation with a synthetic peptide bearing the P101L mutation [25]. While the occurrence of rPrP^Sc^ and sPrP^Sc^ has been extensively investigated in experimental models of GSS P102L, no such studies have been systematically performed in the human disorder. The present study was designed to investigate in eight subjects with GSS P102L the extent of the contribution of PrP^M^ and PrP^Wt^ in the generation of PrP^Sc^ conformers, including sPrP^Sc^ and rPrP^Sc^ molecules. For this purpose, we selected brain tissues from six cases with the 21 kDa and the 8 kDa fragments, either methionine homozygous or heterozygous at codon 129 of the *PRNP*, and two cases harbouring only PrP8, both methionine homozygous. Frozen brain samples were investigated by conventional biochemical techniques, in addition to sensitive protein separation techniques and Western blot with antibodies differentially recognizing the two allelic forms of PrP^C^ and PrP^Sc^. In addition, fixed tissues from the foregoing subjects were investigated by IHC and PET-blot to assess the extent of the contribution of distinct allelic PrP molecules to tissue deposition under different patterns. The results reported here demonstrate (i) a major contribution of PrP^Wt^ to the generation of rPrP^Sc^ species; (ii) the propagation of considerable amounts of mutated and wild-type sPrP^Sc^ molecules; (iii) the extensive tissue deposition and high PET blot signals of both allelic PrP^Sc^ species in all cases, including subjects lacking PrP27-30. Our findings strongly support experimental studies showing a major role of sPrP^Sc^ in causing neurodegeneration in a genetic human prion disease.

## Results

### Immunoblot analyses of protease-resistant PrP^Sc^ in P102L GSS

**Figure 1:**
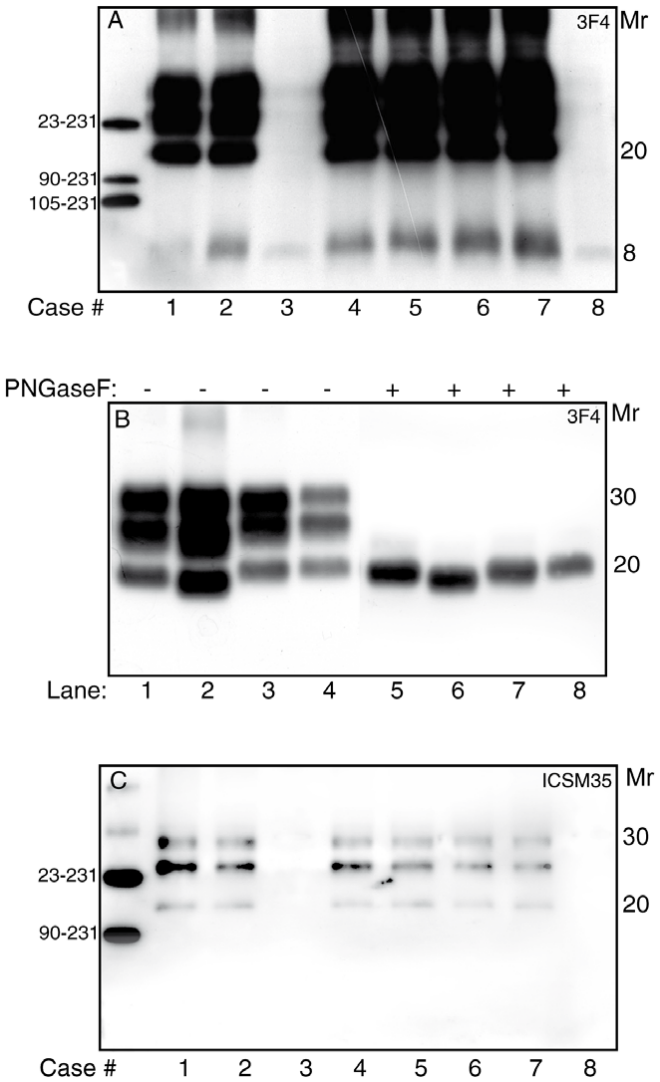
Molecular characterization of mutant and wild-type protease-resistant PrP^Sc^ in GSS P102L. (A) Western blot with the 3F4 antibody of proteinase K-resistant PrP^Sc^ in GSS P102L subjects shows the presence of three bands migrating at 29, 25 and 20 kDa (cases 1–2 and 4–7), in addition to variable amounts of an 8 kDa fragment (cases 1–8). (B) Comparative analysis of PrP27-30, before (lanes 1–4) and after (lanes 5–8) PNGase F treatment, among GSS P102L (lanes 1 and 5), sCJD MV2 subtype (lanes 2 and 6), fCJD E200K (lanes 3 and 7), and sCJD MM1 subtype (lanes 4 and 8). The core fragment of GSS P102L PrP^Sc^ migrates at 20 kDa, in a zone intermediate between type 1 and type 2 PrP^Sc^. (C) Immunoblot analysis of proteinase K-treated GSS P102L brain samples probed with ICSM 35, recognizing wild-type PrP27-30. Molecular mass is shown in kDa.

Demographic, genetic, and clinical data of all investigated subjects are summarized in Table 1. Following treatment with PK and immunoblot with 3F4, frontal cortex homogenates from six GSS P102L subjects showed the presence of three major PrP27-30 bands migrating at 30, 25, and 20 kDa, and a single 8 kDa band; two cases showed only trace amounts of the 8 kDa fragment in the absence of PrP27-30 (Fig. 1A). The migration of the unglycosylated PrP27-30 band was intermediate as compared to type 1 and type 2 sCJD-associated PrP^Sc^, whereas the glycosylation profile showed a relative abundance of the diglycosylated form, similar to fCJD E200K, but at variance with the “monoglycosylated dominant” pattern seen in sCJD brains (Fig. 1B, lanes 1–4). Differences in the migration of PrP27-30 core fragment persisted after glycans removal (Fig. 1B, lanes 5–8), therefore suggesting a common proteolytic cleavage site of glycosylated and unglycosylated PrP^Sc^ species. Cerebellar homogenates were available in four P102L subjects and showed the presence of barely detectable amounts of the 8 kDa fragment in three cases, and of PrP27-30 in one.

**Table 1:** Demographic, molecular, and clinical features of investigated P102L GSS cases.

CASE (code)	Codon 129	Gender	Age at onset	Disease duration	Clinical signs at onset	Symptoms at evolution
1(91-466)	M/V	M	56	60	Ataxia, dysarthria, depression	Dementia
2(92-331)	M/M	M	31	36	Ataxia, dysarthria	Cognitive decline, ataxia, dysarthria, abnormal eye movements
3(96-422)	M/M	M	65	60	Ataxia, dysphasia and dysarthria	Dementia, agitation, sleep disturbance
4(98-196)	M/M	M	57	9	Ataxia, mild cognitive impairment	Cerebellar signs and dementia
5(98-197)	M/V	M	31	11	Confusion	Mental deterioration, apraxia, ataxia
6(2004-59)	M/M	F	41	5	Mild cognitive impairment, behavioral changes, dysarthria, mild ataxia	Abnormal sleep, agitation, dysphasia
7(2005-51)	M/V	M	46	72	Weakness, gait ataxia	Dementia
8(2005-52)	M/M	M	50	36	Cognitive decline	Behavioral disturbances, dementia

Immunoblots probed with ICSM 35 antibody, which fails to recognize human PrP^M^, while detecting PrP^Wt^ [24], showed the presence of PK-resistant PrP^Sc^ in brains from subjects with PrP27-30 (Fig. 1C). In all cases, ICSM 35 failed to detect PrP8, thus confirming the exclusive origin of this internal fragment from the mutated allele. Differently from 3F4-positive PrP27-30, including the total pool of mutated and wild type rPrP^Sc^, ICSM 35-positive PrP27-30 showed a relative abundance of the monoglycosylated forms, and a core fragment migrating at ∼20 kDa. Semiquantitative densitometric analyses showed that wild-type PK-resistant PrP^Sc^ accounted for 5–10% of the total 3F4-reactive PK-resistant PrP^Sc^. Within each case, immunoblots with ICSM 35 and 3F4 showed similar results in brain homogenates from at least three different samples of the frontal cortex.

### Two-dimensional analysis of protease-resistant PrP^Sc^ in P102L brains

**Figure 2:**
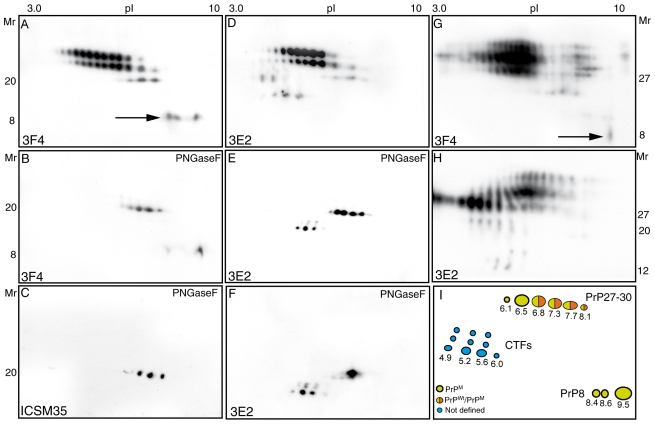
Two-dimensional analysis of mutant and wild-type PrP27-30, C-terminal truncated fragments, and insoluble PrP^Sc^ species in GSS P102L. (A) Immunoblot with 3F4 of PrP27-30 in case # 4 shows three sets of differently glycosylated charge isomers migrating between 30 and 20 kDa, in addition to three spots with a mass of 8 kDa and pIs from 8.4 to 9.6 (arrow). (B) After deglycosylation, the 3F4-reactive core fragment resolves as five spots with net charges between 6.1 and 8.1. (C) ICSM 35 antibody decorates three major spots at 8.1, 7.7, 7.3, and a minor spot at 6.8. (D) Proteinase K-resistant PrP^Sc^ probed with 3E2 shows the three sets of PrP27-30, in addition to acidic isoforms migrating at 20 and 14 kDa, which after deglycosylation (E), resolved as a core fragment of six spots at 20 kDa, and C-terminal truncated species of 16–17- and 12–14-kDa. (F) In contrast, the PrP27-30 core fragment associated to E200K mutation resolves as a major spot at pI of 6.8. Immunoblots of the detergent-insoluble fraction, show the presence of naturally occurring PrP^Sc^ species with migration overlapping PrP27-30, the 8 kDa fragment (G), and C-terminal truncated fragments (H). (I) Schematic diagram of PK-resistant mutant and wild-type PrP^Sc^ forms.

**Figure 3:**
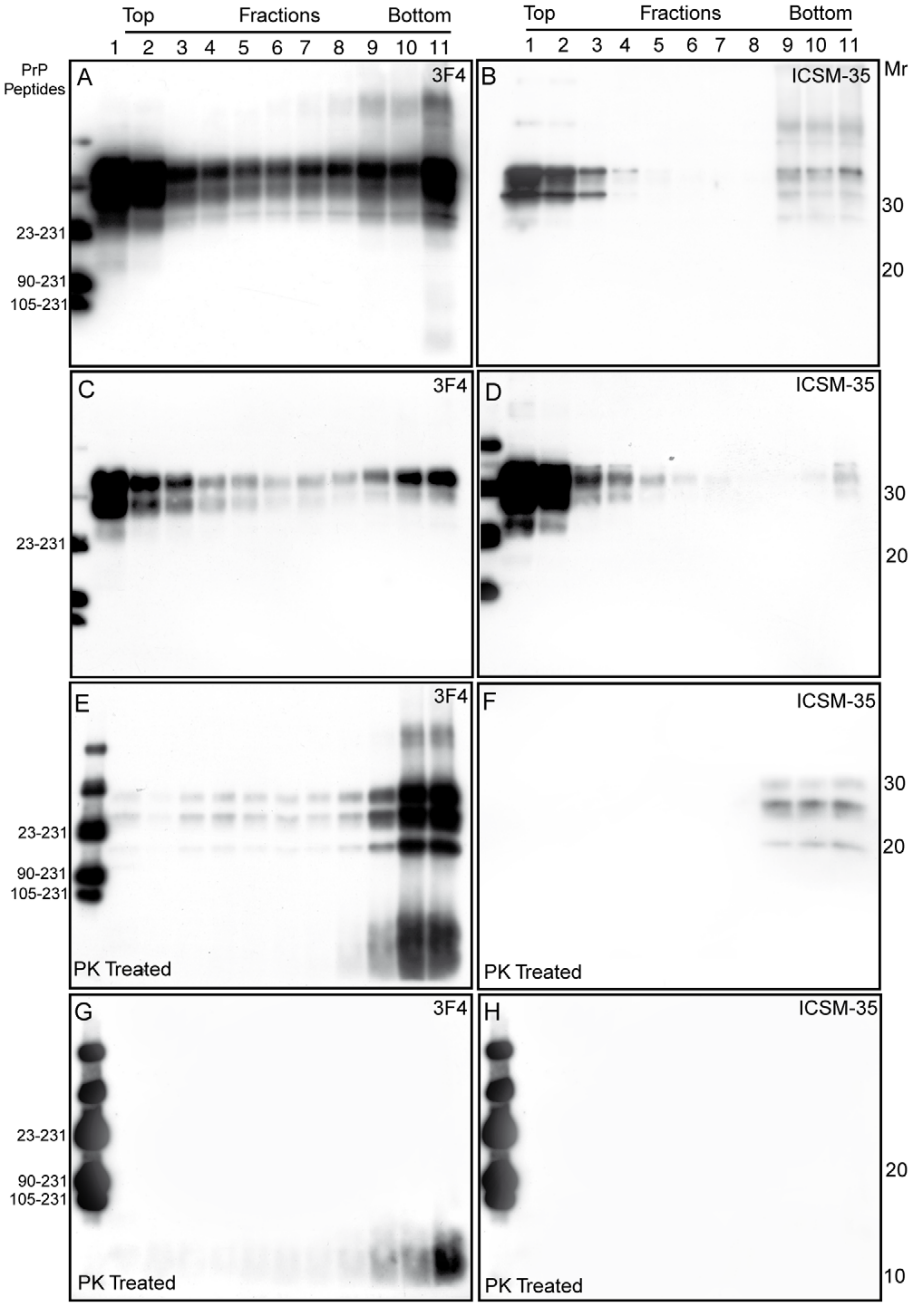
Fractionation of mutant and wild-type PrP^Sc^ aggregates in GSS P102L. Brain homogenates from frontal cortexes of case # 5 (A, B) and case # 8 (C, D) were sedimented in a 10 to 60% sucrose gradient. After sedimentation, half samples were digested with PK (E, F, G, H). (A) PrP from case # 5 is distributed across all fractions albeit concentrated at the top (1–4) and at the bottom fractions (8–11); two bands of 11 and 8 kDa co-localize with PrP^Sc^ in fraction 11. (B) Immunoblot with ICSM-35 of case # 5 shows lower amounts of PrP aggregates, with a distribution similar to that observed with 3F4, in the absence of lower molecular species. (C) In case # 8, 3F4-reactive PrP was mainly distributed at the top and at bottom fractions, whereas ICSM 35 showed very low amount of PrP in the bottom fraction (D). (E) Mutant PrP aggregates in case # 5 were resistant to PK digestion; PK-resistant PrP^Sc^ species were more abundant in bottom fractions, containing also high amounts of the a smear spanning 8–12 kDa. (F) ICSM 35 stained PK-resistant PrP^Sc^ from case # 5 were detected only in the bottom fractions. (G) In case # 8, PrP27-30 is absent, while an 8 kDa fragment is detected at the fractions 9–11; on the contrary, no wild-type PrP species are seen (H).

After 2-D separation and immunoblot with 3F4, PrP27-30 resolved as two sets of glycosylated charge isomers of 30 and 27 kDa, with pIs ranging from 4.0 to 8.0, and three unglycosylated 20 kDa spots, migrating between 8.1-6.8; in addition, three basic spots were seen in the 8 kDa zone (Fig. 2A). After deglycosylation, two additional 3F4-reactive 20 kDa spots, pIs of 6.5 and 6.1, were seen (Fig. 2B), whereas ICSM 35 detected three major spots at 8.1, 7.7, 7.3, and a minor spot at 6.8 (Fig. 2C). Taken together, the PrP^Sc^ core fragment in GSS P102L is composed as follows: (i) PrP^M^ accounts for two major 6.5 and 6.8 spots, and minor 6.1, 7.3, 7.7, and 8.1 charge isomers; (ii) PrP^Wt^ contributes a major 7.7 spot, and two 7.3 and 8.1 charge isomers (Fig. 2I). Immunoblot with 3E2 showed additional spots in the acidic zone of the gel, with an orthogonal migration between 20 and 14 kDa (Fig. 2D); after deglycosylation, the PrP27-30 core fragment resolved as six spots with pIs ranging from 6.1 to 8.1 (Fig. 2E), whereas CTFs of 16–17 kDa and 12–14 kDa were confined to a 4.5–6 range (Fig. 2E). The heterogeneous composition of the core fragment in P102L GSS was not dependent on the glycosylation pattern, since the PrP^Sc^ core fragment in fCJD E200K, which is characterized by a “diglycosylated dominant” pattern, was composed by a single 6.8 spot (Fig. 2F). Collectively, these results show that divergent allelic PrP^Sc^ conformers and glycotypes propagate in GSS P102L. Immunoblots of the detergent-insoluble fraction probed with 3F4 (Fig. 2G) and 3E2 (Fig. 2H), revealed PrP^Sc^ species migrating at 20–30 kDa and 8 kDa, in addition to CTFs of 16–17 and 12–14 kDa, thus demonstrating the occurrence of endoproteolytic products, matching those obtained after PK digestion. Graphic representation of the allelic contribution of PrP^M^ and PrP^Wt^ to PK-resistant PrP species is illustrated in the cartoon (Fig. 2I)

### Sedimentation velocity in sucrose gradients of mutated and wild-type PrP in P102L GSS

As expected, in control human brains, small aggregates of 3F4- and ICSM 35-reactive PrP^C^ species were recovered only in the top fractions. On the contrary, in the six GSS cases with PK-resistant PrP^Sc^, 3F4-reactive species were collected in all gradient fractions, being more abundant in fractions 9 to 11; the latter fractions contained also higher *Mr* PrP aggregates, and, in addition, fraction 11 contained aberrant PrP species of 8–16 kDa (Fig. 3A); fractions stained with mAb ICSM 35 disclosed the presence of small PrP aggregated mainly in the top fractions, in addition to variable amounts of large assemblies in the fractions 9–11, and to barely detectable PrP forms in the intermediate fractions (Fig. 3B). The distribution and association of aberrant PrP molecules in the two cases lacking PK-resistant PrP^Sc^, namely cases # 3 and # 8, provided divergent results, as to the partitioning of 3F4- and ICSM-positive PrP molecules throughout the fractions. Indeed, 3F4-positive PrP molecules were detectable throughout all fractions, being more abundant in fractions 1–3 and 10–11, in the absence of higher molecular and 8–16 kDa species (Fig. 3C); on the contrary, ICSM 35-positive PrP aggregates provided a decreasing signal through fractions 1 to 5 and a weak positivity in the bottom fraction (Fig. 3D). PK-treatment in the six cases with PK-resistant PrP^Sc^ showed the presence of PrP27-30 in all fractions, more abundantly in those reaching the bottom of gradient; the last two fractions also contained high amount of the 8 kDa fragment (Fig. 3E). In these samples, ICSM 35-positive PrP27-30 was seen in fractions 9–11 (Fig. 3F). In cases lacking PrP27-30, PK-resistant PrP species of 8 kDa were decorated by mAb 3F4 in the bottom fractions (Fig. 3G), whereas ICSM 35-positive species were completely degraded by the protease treatment (Fig. 3H).

### Protease-sensitive PrP^Sc^ conformers

**Figure 4:**
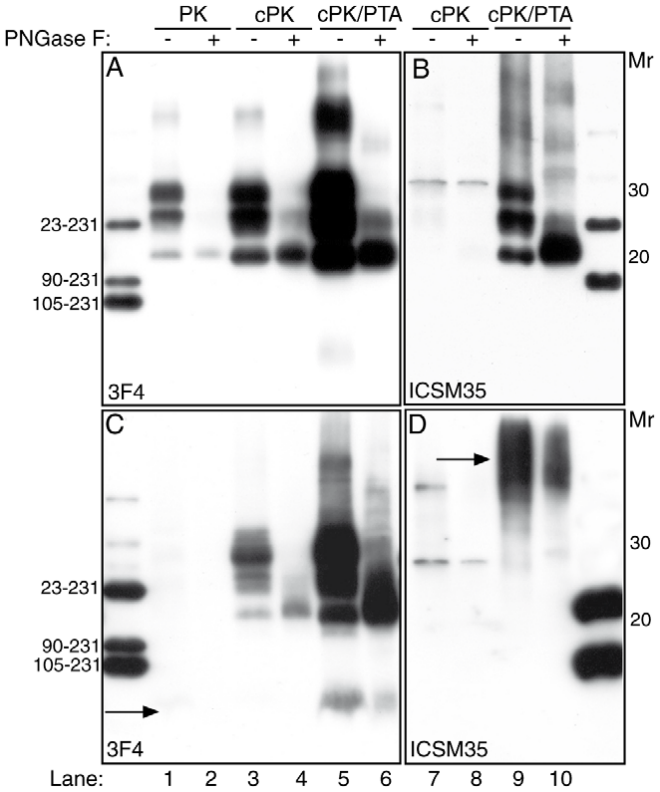
Protease-sensitive PrP^Sc^ conformers in GSS P102L brain tissues. (A) Immunoblot with 3F4 of brain homogenate from case # 2 digested with PK before (lane 1) and after PNGase treatment (lane 2); lanes 3 to 6 show products of ‘cold PK’ (cPK)-treated homogenates, before (lanes 3 and 5) and after (lanes 4 and 6) PNGase treatment; in lanes 5 and 6 samples were NaPTA-precipitated; (B) cPK-treated (lanes 7–10) and NaPTA precipitated products (lanes 9 and 10) were revealed with ICSM 35, prior (lanes 7 and 9) or after deglycosylation. (C) Immunoblot with 3F4 of brain extracts from case # 8, after conventional PK treatment, shows a barely detectable 8 kDa band (lanes 1,2, arrow); in contrast, after cPK digestion (lanes 3 to 6) and NaPTA precipitation (lanes 5 and 6) consistent amounts of PrP27-30 and of the 8 kDa fragment are seen (lane 3–6) in PNGase untreated (lanes 3 and 5) or treated (lanes 4 and 6) preparations. (D) In case # 8, immunoblot with ICSM 35 did not reveal PrP^Sc^ after cPK treatment (lanes 7 and 8), wheras NaPTA precipitation showed a smear migrating in the upper part of the gel in a 50–70 kDa zone (lane 9, arrow), partially decreased by PNFase treatment (lane 10).

Using the “mild PK” protocol, or limited digestion with PK at 4°C, in the six cases with PK-resistant PrP^Sc^ we detected levels of 3F4-positive sPrP^Sc^ higher than amounts of rPrP^Sc^ obtained with the classical “harsh-PK” protocol. The signal of the cold-PK resistant isoforms, including a high *Mr* smear, was greatly enhanced after PTA precipitation, and reduced to a core fragment migrating in a 20–22 kDa zone, after PNGase treatment (Fig. 4A). Immunoblot with ICSM 35 showed that also the wtPrP contributed to the pool of sPrP^Sc^, although at lower levels than the mutated PrP isoform (Fig. 4B). Intriguingly, moderate to high amounts of 3F4-positive sPrP^Sc^ species were recovered in the two cases lacking the 21 kDa fragment (Fig. 4C), in addition to ICSM 35-positive high *Mr* sPrP^Sc^ species (Fig. 4D, arrow). In all investigated subjects, sPrP^Sc^ species included the 8 kDa fragment, which was detected by the 3F4 antibody, but not the ICSM 35 (Table 2). Collectively, these findings are at variance with results obtained with GSS^P101L^ prions in transgenic mice, which lack PrP8 and have a sPrP^Sc^ core fragment of 22–24 kDa [14]. Brain homogenates from frontal cortex of GSS P102L subjects with and without PrP27-30, digested with increasing concentrations of PK, ranging from 5 to 50 µg/ml, and probed with 1E4, did not show the ladder-like pattern observed in subjects with “variably protease-sensitive prionopathy” (VPSP) and GSS A117V [26].

**Table 2:** Allelic derivation and semiquantitative analysis of protease-resistant and protease-sensitive pathological prion protein species in P102L GSS cases.

Case (code)	Conventional PK treatment(100 µg/ml 37°C)				Cold PK treatment(250 µg/ml 4°C)			
	3F4		ICSM-35		3F4		ICSM-35 (PTA)	
	PrP27-30	8 kDa	PrP27-30	8 kDa	PrP27-30	8 kDa	PrP27-30	8 kDa
1(91-466)	+++	+++	+/−	−	+++	+++	+++	−
2(92-331)	++	++	+/−	−	++	++	+++	−
3(96-422)	−	++	−	−	++	+	−	−
4(98-196)	++	+	+/−	−	++	+	+++	−
5(98-197)	+++	+	+/−	−	+++	+	+++	−
6(2004-59)	+++	+	+/−	−	+++	+	+++	−
7(2005-51)	+++	++	+/−	−	++	++	−(*)	−
8(2005-52)	−	+	−	−	++	++	+/−	−

(*)in the upper part of the gel a smear is observed (see figure 5D). Scores: − (negative); +/− (faint); + (weak); ++ (moderate); +++ (intense).

### PrP IHC

In all investigated subjects, tissue deposition of PrP occurred as different patterns, including synaptic-like, granular, diffuse, unicentric and multicentric plaques (Fig. 5A–F, Table 3). We also noted a fine granular type of PrP deposition in the white matter, suggestive of an axonal location. Plaques were labeled by 3F4 (Fig. 5A,5B,5C) and ICSM 35 (Fig. 5D,5E,5F) antibodies, and, as far as individual plaques could be identified in consecutive paraffin- or plastic-embedded sections (data not shown), we were unable to observe ICSM 35-negative plaques. On the contrary, diffuse and synaptic-like PrP deposits were exclusively labeled by 3F4, but not ICSM 35 antibody. Noteworthy, the use of antibody directed against the N- and C-terminus disclosed a peripheral staining of plaques (data not shown). PrP immunostaining in case # 3 was indistinguishable from that observed in GSS cases with PrP27-30 (Fig. 5G,5H). Conversely, in case # 8 an atypical pattern of PrP deposition was seen within all cortical layers of the frontal cortex. IHC with 3F4 showed synaptic-type positivity and “filamentous” PrP aggregates in a wavy pattern of deposition (Fig. 5I,5J). In this case, packed PrP deposits were similar to the “curly” pattern recently described by Colucci et al. in GSS H187R coupled with V at codon 129 (H187R-129V haplotype) [27]; IHC with ICSM 35 revealed the association of fibrillary prion protein aggregates with glial cell membranes. Additionally, punctuate PrP deposition was observed in the white matter with both antibodies. Taken together, in the two cases lacking PrP27-30 at immunoblot, deposition of mutated and wt PrP was observed under diverging patterns, either as granular and plaque deposits, or in a wavy motif. This further confirms that sPrP^Sc^ is involved into mechanisms of aggregation and tissue deposition.

**Figure 5:**
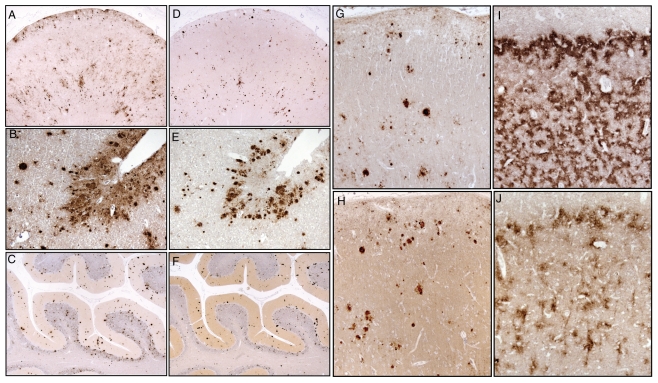
Mutant and wild-type PrP immunostaining in GSS P102 L. IHC with the 3F4 antibody shows PrP deposition under diffuse, synaptic-like, and plaques aggregates in the frontal cortex (A, B) and in the cerebellar cortex (C) of case # 3; IHC with ICSM 35 in adjacent sections of the same subject immunodecorates PrP plaques in both frontal cortex (D, E) and cerebellum (F), but not diffuse or synaptic-like PrP deposits. (G) Frontal cortex from case # 3 showing 3F4-positive plaques, as well as of granular/synaptic-like PrP deposits; the punctuate and granular patterns are less evident with the ICSM 35 antibody (H), whereas a similar stain is obtained for plaques. (I) 3F4 immunostaining of the frontal cortex from case # 8 showing a widespread PrP deposition involving all cortical layers; the PrP pattern of deposition is “curly-like”; (J) immunostaining with ICSM 35 in an adjacent section shows a curly PrP deposition in the outer layer of cortex as well as a pericellular staining.

**Table 3:** Immunohistochemical analysis of allelic prion protein species in P102L GSS.

Case (code)	Frontal Cortex		Cerebellum	
	3F4	ICSM 35	3F4	ICSM 35
1(91-466)	++++Multicentric cortical and subcortical plaques; granular cortical staining. Axonal staining (Fig. 3A, 3C, 3G)	++As with 3F4, but less intense granular and axonal deposition (Fig. 3B, 3D, 3H)	++++Amyloid plaques; granular deposition in the granular layer (Fig. 3E)	+++Granular and axonal staining (Fig. 3F)
2(92-331)	+++Diffuse staining and multicentric plaques	++Weaker reactivity of plaque core, as compared to 3F4	+++Diffuse staining and multicentric amyloid plaques	++Weaker staining of plaque core
3(96-422)	++++Granular deposition, multicentric amyloid plaques (Fig. 3 I)	++As with 3F4, but less intense granular and axonal deposition (Fig. 3J)	++++Amyloid plaques in molecular and granular layers; axonal deposition	++Weaker staining of amyloid plaques
4(98-196)	N.A.	N.A.	N.A.	N.A.
5(98-197)	N.A.	N.A.	N.A.	N.A.
6(2004-59)	+++Diffuse plaques, granular deposition on the whole cortex. Plaques and axonal staining in the white matter	++As with 3F4, except for reduced granular and axonal staining	++++Numerous multicentric amyloid plaques involving the whole cerebellum. Axonal staining	++Granular and axonal deposition
7(2005-51)	+++Granular staining and amyloid plaques	++As with 3F4, except for the absence of granular staining	N.A.	N.A.
8(2005-52)	++++“Curly-like” pattern in the cerebral cortex. Intense axonal staining in the white matter (Fig. 3K and 3M)	++Diffuse staining (Fig. 3L and 3N)	+Synaptic-type deposition in the molecular layer	−

N.A. Not available. Scores: − (negative); + (light); ++ (mild); +++ (moderate); ++++ (intense).

### PET-blot detection of abnormal PrP species

**Figure 6:**
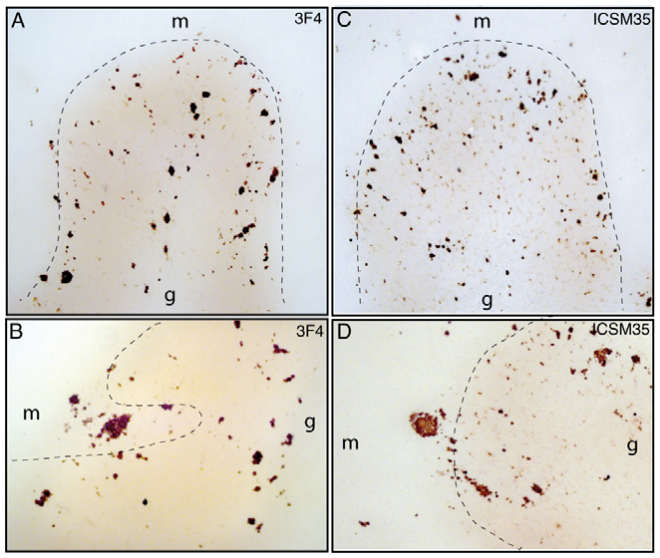
Paraffin-embedded tissue-blot analysis of mutant and wild-type PrP. Detection of proteinase K-resistant forms of mutant (A, B) and wild-type (C, D) prion protein in the cerebellum of case # 1, as decorated by 3F4 and ICSM 35 antibodies; g denotes the granular layer, and m denotes the molecular layer.

PET-blot with 3F4 (Fig. 6A,6B) and ICSM 35 (Fig. 6C,6D) was performed on GSS P102L brain tissues samples from the frontal cortex and cerebellum, in corresponding brain regions investigated by IHC. PrP deposition under granular and plaque aggregates was identified with both antibodies in all investigated frontal and cerebellar sections, including cases with and without PrP27-30; no PrP deposition was detectable in the tissues of the non-prion control group. The immunolabeling obtained with the PET blot method showed a pattern similar to that observed with IHC, with the exception of the synaptic pattern, which could not be clearly assessed owing to the resolution limitations of sections examined with this technique. To date, PET-blot has been performed only in one GSS case [28], but not with antibodies recognizing PrP^wt^, and, more importantly, not in cases lacking PK-resistant PrP^Sc^. Therefore, the present results, have important implication for the diagnostic role of this methodic in prion diseases.

## Discussion

**Figure 7:**
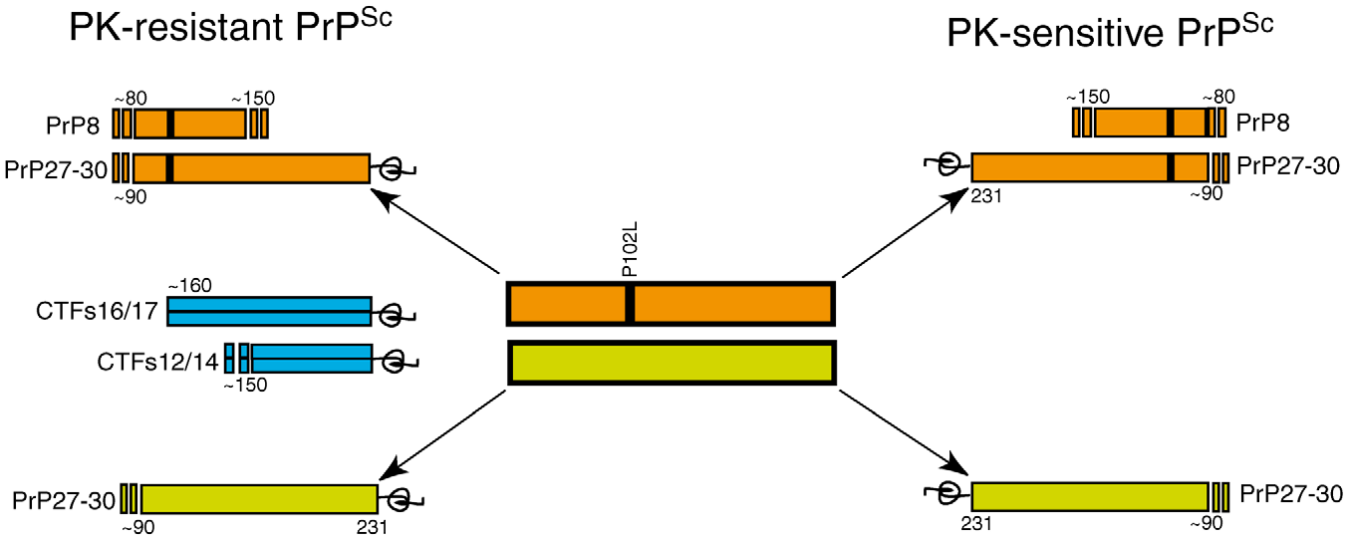
Diagram of PK-resistant and PK-sensitive PrP^Sc^ molecules detected in GSS with the P102L mutation. PrP^Sc^ species originating from the mutant allele are depicted in orange, whereas wild-type PrP^Sc^ molecules are depicted in green. PrP8 is an internal PK-resistant fragment spanning residues 74/90 to 146/153. PrP27-30 is the main C-terminal PK-resistant PrP fragment generated by PK cleavage between PrP^Sc^ residues 74 and 103. PrP-CTF 16/17 and PrP-CTF 12/13 are smaller PK-resistant fragments whose allelic origin remains undetermined (depicted in blue).

The results reported here show that the heterogeneity of PrP^Sc^ species associated with GSS P102L mutation is wider than previously reported and is characterized by different degree of involvement of PrP^M^ and PrP^Wt^ (Fig. 7). Our findings also provide important clues as to the relative contribution of allelic PrP molecules to neuropathological changes and tissue accumulation of PrP and, in turn, on the sensitivity of IHC and PET blot in detecting pathological forms of prion protein.

We show that the composition of rPrP^Sc^ forms in GSS P102L is more heterogeneous than previously recognized, since, in addition to PrP27-30 and PrP8, discrete amounts of CTFs with an intermediate migration at 16–17 kDa and 12–14 kDa were found. Using conventional and sensitive protein separation techniques, we confirm the different glycotype between rPrP^M^ and rPrP^Wt^ PrP27-30 [24], and, moreover, we show that rPrP^M^ and rPrP^Wt^ have distinct conformations. These conclusions are highlighted by the heterogeneous composition of PrP27-30 core fragment, including distinct 3F4- and ICSM 35-positive PrP^Sc^ charge isomers, whose characterization was not possible under conventional Western blot [24]. This hybrid pattern, reflecting the distinct contribution of rPrP^M^ and rPrP^Wt^, was independent from polymorphic codon 129 since it was observed in MM and MV subjects. In addition, the 3F4-positive core fragment was at variance with fCJD E200K, sharing a diglycosylated dominant pattern of PrP27-30 with GSS P102L, therefore ruling out any influence of the glycosylation status. The combined contribution of PrP^M^ and PrP^Wt^ to PrP27-30 species is likely to account for the molecular complexity of pathological prion protein in GSS P102L cases and for its influence into the phenotypic variability throughout propagation of distinct and multiple molecules.

The detection of CTF16-17 in GSS P102L subjects with PrP27-30 speaks in favor of an aberrant endogenous proteolytic pathway of PrP^Sc^, as suggested by their detection in the native insoluble fraction, and provides molecular support to similarities with sCJD with type 1 PrP^Sc^ and MM2 subtype [6]. On the contrary, the significance of CTF12-14 is less clear, since these species have been detected in all molecular types of sCJD; their presence in GSS P102L cases is intriguing, given the strict contiguity of the N-terminus of CTF12-14 at amino acid 154–167 with the C terminus of PrP8 at 146–153. Zou et al. previously observed that, in sCJD, CTF12-14 likely originate from a subpopulation of PrP^Sc^ distinct from the population which generates PrP27-30 [29]; in addition, due to their inability to detect PrP8 in preparations containing CTF12-14, these authors considered unlikely the possibility that two separate cleavages of the full-length PrP^Sc^ could result in the generation of PrP-CTF12-14 and PrP8. The hypothesis that PrP8 and CTF12-14 are generated from different populations of PrP^Sc^ is further strengthened by our findings showing the failure to detect CTF12-14 in cases expressing only PrP8. Taken together, our present results support the view that in GSS P102L PrP27-30, PrP8, CTF16-17, and CTF12-14 species are generated by multiple and distinct conformational pools of protease-resistant PrP^Sc^, likely originating from diverging subsets of protease-resistant molecules. Although the allelic origin of N-terminally truncated fragments in GSS could not be assessed, we argue that CTFs may originate from both PrP^M^ and PrP^Wt^.

Over the past few years, it has been shown that several prion strains harbor protease-resistant and protease-sensitive PrP^Sc^ molecules, and also that some human genetic and sporadic conditions are characterized by the exclusive presence of protease-sensitive PrP^Sc^ conformers. Further, a number of observations have suggested that molecular species other that protease-resistant PrP may be involved in the pathogenesis of GSS [12], [13], [14], [15]. Hedge et al. first provided evidence that brain tissues from two GSS subjects with the A117V mutation and from Tg mice expressing various *PRNP* mutations had increased levels of ^CTM^PrP, a topological isoform of PrP^C^, in the absence of protease-resistant PrP^Sc^ [30]. In addition, these authors provided experimental evidence about the central role of ^CTM^PrP in inducing neurodegeneration. The detection of neurotoxic PrP isomers was possible thanks to the “cold PK” digestion, an assay conceived to detect subtle conformational differences in proteins, taking advantage of their differential sensitivity to a weakly active protease. This alternative method was more recently employed to detect pathological conformers of the prion protein, other than PK-resistant PrP^Sc^, in transgenic mice expressing the equivalent human P102L GSS mutation at codon 101 (P101L), which develop spontaneous prion disease, as well as in Tg mice inoculated with brain homogenates from GSS P102L patients expressing valine or methionine at codon 129, and in the brain of a P102L M129 GSS case [14]. Collectively, the foregoing experiments have provided evidence that in the P102L mutation alternative misfolding pathways take place, inducing the formation of abnormal conformers other than PK-resistant PrP^Sc^, which can revealed by the “cold PK” assay. However, one biochemical feature of these experimental studies is the striking absence of PrP8, either among protease-resistant PrP species or sPrP forms, and the lack of information regarding the allelic derivation of distinct abnormal PrP species.

In this study, the issue of sPrP^Sc^ and rPrP^Sc^ in P102L was investigated, after the evidence of different pools of PrP^Sc^ with variable distribution in sedimentation profile, and diverging resistance to conventional PK treatment. We introduced PK digestion in “cold condition” to preferentially detect sPrP^Sc^ species, in parallel to the canonical PK digestion at 37°C which retrieves rPrP^Sc^ molecules. Obtained results confirm earlier experimental findings in Tg mice on the presence of significant levels of “cold PK-resistant” PrP^Sc^ in all cases. Under the foregoing experimental conditions we confirm that sPrP^Sc^ from the mutated and wt alleles generate fragments with molecular weight similar to those of rPrP^Sc^, an issue not investigated by Wadsworth and collaborators [24]. Noteworthy, cold PK sensitive PrP27-30 e PrP8 were found also in cases lacking rPrP^Sc^, and they were highly retrieved by PTA precipitation. The biochemical properties of P102L sPrP^Sc^ species were distinct from molecular PrP forms encountered in GSS A117V and in VPSPr [26]. These findings indicate that different human prion diseases are characterized by the generation of multiple conformational quasispecies of sPrP^Sc^ [31].

Despite the wide use of PrP IHC in the diagnosis of prion diseases, it is still unclear which molecular forms of PrP^Sc^ are detected by IHC after formic acid treatment and hydrolytic autoclaving, or even after PK treatment; this represents a major limitation in using IHC as a diagnostic tool in prion diseases. It has been argued that autoclaving of tissue sections, treated with formic acid, might destroy only PrP^C^ and sPrP^Sc^ (also lost after limited proteolysis with PK), but not rPrP^Sc^ that is only denatured. However, this explanation is in contrast with our present IHC and PET blot results, showing PrP deposition in cases lacking rPrP^Sc^ species. Therefore, the diagnostic sensitivity of IHC and PET blot as compared with immunoassay, needs to be reassessed in prion diseases, also taking account of the fixation protocol. According to our present results, PrP^M^ contributes to synaptic-type staining, whereas both PrP^wt^ and PrP^M^ concur to granular, axonal, and amyloid PrP deposition. Further, PET blot analysis with both 3F4 and ICSM35 showed a comparable staining of PrP plaques, in sections that were stained following PK digestion. To explain the present results we suggest that fixation with formalin may induce protein-protein covalent cross links, owing to reactivity of formaldehyde with side chain moieties of lysyl, arginyl, tyrosyl, aspartyl, histidyl, and seryl residues. These changes might induce sPrP^Sc^ to acquire resistance to chemical and physical treatments, including protease digestion. This line of thinking is readily evident when one considers that cases bearing only the internal PrP8 fragment, in the absence of 3F4- and ICSM 35-positive PrP27-30, showed marked deposition of PrP^Wt^. The present findings lend support to results obtained in GSS H187R-129V, showing intense PrP deposition under “curly” plaques in spite of minimal amount of rPrP^Sc^ [27], and expand the range of GSS mutations associated with curly-like sPrP^Sc^ deposition.

The present findings widen the spectrum of propagating PrP^Sc^ isoforms in P102L GSS, thus providing further molecular support to explain the ample phenotypic heterogeneity observed in this inherited condition.

## Materials and Methods

### Ethics statement

All studies and procedures were done under protocols approved by the Institutional Review Board at Indiana University. The brains of all subjects included in the study were obtained after the families had expressed a strong wish that we study them neuropathologically and carry out research. The brains were harvested with full cooperation of the pathologists involved and following the family's written informed consent.

### Patients and tissue samples

The eight subjects analyzed in the present study were from four unrelated kindred (Table 1). Pedigrees, clinical features, neuropathological and molecular data of 5 cases (91-466, 92-331, 96-422, 98-196, 98-197) have been previously reported. Frozen and formalin-fixed brain tissues from all patients were used for the present study. Control brains were obtained from subjects not affected by prion diseases. The use of stored brain tissues in this study was approved by the ethical committee.

### Genetic analysis

Analysis of *PRNP* was performed by using genomic DNA extracted from frozen brain tissue. Amplification of the *PRNP* coding region was obtained by polymerase chain reaction; the codon 129 genotype and the codon 102 mutation were determined by restriction enzyme analysis, as previously described [9]. *PRNP* sequence analyses showed the presence of allelic P102L point mutation coupled with methionine at codon 129 in all cases. Five patients were methionine homozygous at *PRNP* codon 129, whereas three patients were heterozygous.

### Immunoblot analysis

Brain samples were homogenized in 9 volumes of lysis buffer (100 mM sodium chloride, 10 mM EDTA, 0.5% Nonidet P-40, 0.5% sodium deoxycholate, 10 mM Tris, pH 7.4) and clarified by centrifugation at 1,000×*g* for 10 min. The supernatant (S1) was stored at −80°C until use and the pellet discarded. S1was centrifuged at 100,000*g* for 90 min at 4°C. The supernatant (S2) was saved and the pellet was resuspended in lysis buffer and centrifuged as above. The final pellet (P3) was designated as detergent-insoluble, whereas the detergent-soluble fraction was referred to the final combination of S2 and S3. Protease resistance was assayed by incubating sample aliquots containing 40 µg of total protein with 100 µg of proteinase K (Boehringer Mannheim, Germany) per milliliter at 37°C for 60 minutes. For “cold PK” digestion, clarified 10% brain homogenates containing 1% NP-40 were incubated with 250 µg of PK/ml for 1 h on ice and thereafter treated according to the protocol of Tremblay et al. [14]. The reaction was stopped by the addition of protease inhibitor (5 mM phenylmethylsulfonyl fluoride). Phosphotungstate (PTA) precipitation, after cold PK digestion, was done as previously described [32]. For N-deglycosylation, samples were treated with *N*-glycosidase F (PNGase F) according to the manifacturer's instructions (Boehringer, Mannheim) for 8 hours at 37°C. Proteins were dissolved in sample buffer (3% SDS, 3% β-mercaptoethanol, 2 mM EDTA, 10% glycerol, 62.5 mM Tris, pH 6.8) and boiled for 5 minutes. An equivalent of 0.4 mg of wet tissue was loaded on 12% SDS-PAGE gels and proteins transferred onto PVDF membrane (Immobilon P, Millipore) for 2 hours at 60 V. Membranes were blocked with 1% non-fat dry milk in TBST (10 mM Tris, 150 mM NaCl, 0.1% Tween-20, pH 7.5) for 1 hour at 37°C and incubated overnight at 4°C with the following anti-human PrP monoclonal antibodies: 3F4 recognizing human PrP residues 106–110 (1∶10.000), ICSM 35 whose epitope spans residues 93–105 and specifically detects wild-type but not P102L human PrP, 6H4 directed to residues 144–152 (Prionics, 1∶5000), 3E2 directed to human PrP residues 214 to 231 (1∶500) (kindly donated by Dr. Capucci), and mouse monoclonal antibody 1E4 against human PrP residues 97–108 (Cell Sciences, Inc., Canton, MA). Blots were developed with an enhanced chemiluminescence system (ECL, Amersham) and PrP visualized on autoradiography films (Hyperfilm, Amersham). Films were scanned by using a densitometer (GS-200, Biorad).

### Two-dimensional gel electrophoresis

For isoelectric focusing (IEF), using immobilized pH gradients (IPG) in the first dimension, pre-cast gels with a linear pH range of 3–10 were used (Biorad). Before IEF, the dry gels were reswollen for 14–15 hours in 125 µl of buffer (6 M urea, 2 M thiourea, 5% β-mercaptoethanol, 2% Nonidet P-40, and 2% ampholyte) containing an equivalent of 2 mg of wet tissue. IEF was carried out at 20°C for 4 hours with raising voltage (500–8000 V), in a cooled horizontal electrophoresis unit (IPGphor, Pharmacia). For the second dimension, the IPG strips were equilibrated for 20 minutes in 50 mmol/L Tris-HCl, 6 M Urea, 10% glycerol, 2% SDS and a trace of bromophenol blue and loaded on a 16% SDS-PAGE. Immunoblotting was performed as described above and PrP was revealed with 3F4 (1∶10.000, Dako), 3E2 (1∶500) and ICSM 35 (1∶1000, D-Gen).

### Sedimentation velocity in sucrose gradient

The S1 fractions prepared by centrifugation of 20% brain homogenates at 8000 *g* for 5 min at 4°C were incubated with an equal volume of 2% Sarkosyl for 30 min on ice. The samples were loaded atop a 10–60% step sucrose gradient and centrifuged at 200,000 *g* for 1 h at 4°C, as described by Tzaban et al. [33]. Eleven fractions were collected from the top of the tube for Western blot analysis of PrP.

### PrP immunohistochemistry

Paraffin sections that were 8 µm thick were deparaffinized, rehydrated in descending graded alcohols, treated with 98 percent formic acid for 20 minutes at room temperature, and autoclaved at 121°C for 10 minutes in 1.5 mM hydrochloric acid. Sections were rinsed and then incubated overnight at 4°C with monoclonal antibody 3F4 (1∶500 dilution) and ICSM 35 (1∶250). Subsequent antibody detection involved incubation with a biotinylated goat anti-mouse secondary antibody for one hour (1∶500 dilution, Vector Laboratories) at room temperature, followed by incubation with the avidin–biotin–peroxidase complex (Vectastain ABC-Elite kit, Vector Laboratories) according to the manufacturer's instructions. The samples were then stained with 0.06 percent 3,3′-diaminobenzidine as the chromogen and 0.006 percent hydrogen peroxide in 50 mM Tris buffer, pH 7.6.

### Paraffin-embedded tissue blot (PET-blot)

Paraffin sections, 7 mm thick, were collected on prewetted 0.45 µm-pore nitrocellulose membrane and dried for 30 minutes. The sections were thereafter deparaffinized, rehydrated, and treated with 250 µg/ml of proteinase K for 4 h at 37°C. Sections were incubated with monoclonal antibodies 3F4 and ICSM 35. After washing in TBST, incubation with an alkaline phosphatase-coupled rabbit anti-mouse antibody (Dako) was done. The membranes were then adjusted to alkaline pH by exposure to NTM (100 mmol/L Tris-HCl, pH 9.5; 100 mmol/L NaCl; 50 mmol/L MgCl_2_). The visualization of the antibody reaction was provided by formazan reaction using NBT/BCIP. Blots were evaluated with an Olympus microscope.
